# PReS13-SPK-1034: looking for new monogenic forms of lupus

**DOI:** 10.1186/1546-0096-11-S2-I3

**Published:** 2013-12-05

**Authors:** Y Crow

**Affiliations:** 1 Genetic Medicine, University of Manchester, Manchester, UK

## 

We can expect that a better understanding of the pathogenesis of systemic lupus erythematosus (SLE) will eventually lead to improved treatments for this devastating disorder ('translational medicine'). And if lupus is not a single disease entity, then different treatment regimens might be appropriate in different patients ('personalized medicine').

As one approach to improving our understanding of why some people develop lupus, we are interested in the identification and analysis of monogenic (i.e. Mendelian) forms of SLE - thinking that they will not only provide clues to disease causation in specific cases, but that they will also help to define general molecular concepts of immune tolerance/dysfunction in humans, and thus inform the experimental approaches of other researchers (by highlighting potentially important disease pathways e.g. the 'type I interferonopathies').

Drawing on a well-proven strategy taken in other 'complex diseases' (e.g. certain types of cancer, motor neuron disease, Parkinson disease, Alzheimer disease etc.), we hypothesise that populations of children affected by lupus will likely be enriched for Mendelian forms. We are therefore harnessing the power of next-generation sequencing technology to identify highly penetrant genetic susceptibility loci for juvenile SLE. In this talk, I will discuss our strategy, outline the challenges involved in such an approach, and highlight some recent successes.

## Disclosure of interest

None declared.

